# Utilization and acceptance of virtual patients in veterinary basic sciences – the vetVIP-project

**DOI:** 10.3205/zma001096

**Published:** 2017-05-15

**Authors:** Christin Kleinsorgen, Marta Kankofer, Zbigniew Gradzki, Mira Mandoki, Tibor Bartha, Maren von Köckritz-Blickwede, Hassan Y. Naim, Martin Beyerbach, Andrea Tipold, Jan P. Ehlers

**Affiliations:** 1 University of Veterinary Medicine Hannover, Foundation, E-Learning Department, Hannover, Germany; 2 University of Life Sciences Lublin, Faculty of Veterinary Medicine, Department of Biochemistry, Lublin, Poland; 3 University of Life Sciences Lublin, Faculty of Veterinary Medicine,Department of Epizootiology and Clinic of Infectious Diseases, Lublin, Poland; 4 Szent István University, Veterinary Faculty, Department of Pathology and Forensic Veterinary Medicine, Budapest, Hungary; 5 Szent István University, Veterinary Faculty, Department of Physiology and Biochemistry, Budapest, Hungary; 6 University of Veterinary Medicine Hannover, Foundation, Department of Physiological Chemistry, Hannover, Germany; 7 University of Veterinary Medicine Hannover, Foundation, Institute for Biometry, Epidemiology and Information Processing, Hannover, Germany; 8 University of Veterinary Medicine Hannover, Foundation, Small Animal Clinic, Hannover, Germany; 9 University Witten-Herdecke, Didactics and Educational Research in Health Science, Witten, Germany

**Keywords:** veterinary education, educational activities, virtual systems, CASUS-software

## Abstract

**Context: **In medical and veterinary medical education the use of problem-based and cased-based learning has steadily increased over time. At veterinary faculties, this development has mainly been evident in the clinical phase of the veterinary education. Therefore, a consortium of teachers of biochemistry and physiology together with technical and didactical experts launched the EU-funded project “vetVIP”, to create and implement veterinary virtual patients and problems for basic science instruction. In this study the implementation and utilization of virtual patients occurred at the veterinary faculties in Budapest, Hannover and Lublin.

**Methods: **This report describes the investigation of the utilization and acceptance of students studying veterinary basic sciences using optional online learning material concurrently to regular biochemistry and physiology didactic instruction. The reaction of students towards this offer of clinical case-based learning in basic sciences was analysed using quantitative and qualitative data. Quantitative data were collected automatically within the chosen software-system CASUS as user-log-files. Responses regarding the quality of the virtual patients were obtained using an online questionnaire. Furthermore, subjective evaluation by authors was performed using a focus group discussion and an online questionnaire.

**Results: **Implementation as well as usage and acceptance varied between the three participating locations. High approval was documented in Hannover and Lublin based upon the high proportion of voluntary students (>70%) using optional virtual patients. However, in Budapest the participation rate was below 1%. Due to utilization, students seem to prefer virtual patients and problems created in their native language and developed at their own university. In addition, the statement that assessment drives learning was supported by the observation that peak utilization was just prior to summative examinations.

**Conclusion: **Veterinary virtual patients in basic sciences can be introduced and used for the presentation of integrative clinical case scenarios. Student post-course comments also supported the conclusion that overall the virtual cases increased their motivation for learning veterinary basic sciences.

## Zusammenfassung

**Hintergrund: **Die Anwendung von problembasierter und fallbasierter Lehre ist in der medizinischen sowie tiermedizinischen Ausbildung in den letzten Jahren angestiegen. An einigen tiermedizinischen Einrichtungen zeigt sich diese Entwicklung vornehmlich im klinischen Teil der veterinärmedizinischen Ausbildung. Daher hat eine Arbeitsgruppe, bestehend aus Dozierenden der Biochemie und Physiologie zusammen mit technischen und didaktischen Experten das EU-finanzierte Projekt „vetVIP“ ins Leben gerufen, um tiermedizinische virtuelle Patienten und Probleme für die Lehre der Grundlagenfächer zu erstellen und zu implementieren. In dieser Projektstudie wurden sowohl die Implementierung, als auch die Nutzung und Anwendung von virtuellen Patienten an den tierärztlichen Fakultäten in Budapest, Hannover und Lublin untersucht.

**Material und Methoden:** Dieser Artikel beschreibt Untersuchungen zur Nutzung und Akzeptanz eines optionalen Zusatzangebotes in Form von online Lernmaterialen an Studierenden im tiermedizinischen Grundlagenstudium, welches begleitend zu den regulären Lehrveranstaltungen in den Fächern der Biochemie und Physiologie angeboten wurde.

Die Reaktion der Studierenden auf dieses Angebot von klinisch fallbasierter Lehre in den Grundlagenwissenschaften wurde anhand quantitativer und qualitativer Daten analysiert. Zum einen wurden quantitative Daten innerhalb des gewählten Software-Systems CASUS als Nutzer-Logbuch-Dateien automatisch gesammelt. Zum anderen wurden Rückmeldungen in Bezug auf die Qualität der virtuellen Patienten mit Hilfe eines Online-Fragebogens erhoben. Darüber hinaus wurde die subjektive Bewertung durch die Fallautoren anhand einer Fokusgruppendiskussion sowie anschließenden Online-Befragung durchgeführt.

**Ergebnisse: **Sowohl die Implementierung, als auch die Nutzung und Akzeptanz variierte an den beteiligten drei Standorten. Eine hohe Zustimmung wurde in Hannover und Lublin anhand der hohen freiwilligen Beteiligung von Studierenden (>70%), welche die optionalen virtuellen Patienten genutzt haben, dokumentiert. Wohingegen die Beteiligung in Budapest unter 1% lag. Aufgrund der Nutzungsdaten lässt sich ableiten, dass die Studierenden virtuelle Patienten und Probleme bevorzugen, die in ihrer Muttersprache geschrieben und an ihrer eigenen Universität entwickelt wurden. Zusätzlich wurde der „Assessment Drives Learning“-Effekt durch die Beobachtung gestützt, dass die Spitzenauslastung der Fallbearbeitungen kurz vor den Abschlussprüfungen im Fach Biochemie lag.

**Schlussfolgerung: **Veterinärmedizinische virtuelle Patienten können bereits in den Grundlagenwissenschaften eingeführt und für die Darstellung integrativer klinischer Fallszenarien verwendet werden. Anmerkungen der Studierenden nach dem Kurs unterstützen die These, dass der Einsatz von virtuellen Fallbeispielen insgesamt die Motivation für das Lernen der veterinärmedizinischen Grundlagenwissenschaften steigert.

## 1. Introduction

From biochemists’ and educationalists’ point of view, the teaching of basic sciences is not only a mandatory legal formality [https://www.gesetze-im-internet.de/tappv/], [1], but also a necessary foundation of basic knowledge and understanding for future clinical thinking and diagnostic skills [2], [3]. The challenge is to establish content understanding as well as to motivate the students to pursue lifelong learning [4], [5], [6]. 

Similar approaches to preclinical veterinary education were already described a decade ago: Ryan et al. (2003) stated that *“various teaching methods already implemented in preclinical veterinary program, were considered useful to the deep approach to learning”, and “The prevalent perception of a high workload is notable, as is its positive association with surface learning.”* [7]. Strategies which likely would reduce the tendency for surface learning and alleviate ‘fear of failure’ were suggested in Ryan’s article. Providing context for learning and understanding, by using integration between basic and clinical knowledge, further arousing attention, increasing active learning and aligned with personal interests, is a valuable tool for intrinsic motivation and intellectual satisfaction of students during the process of studying [6]. Hence, case-based learning (CBL) and problem-based learning (PBL) have been widely introduced in veterinary medical education to expose students to real clinical problems to arouse their interest and enhance the ability for clinical and diagnostic thinking skills.

Another trend is the use of virtual patients (VPs). Previous studies confirmed the use of e-learning and VPs as efficient and auspiciously demonstrated better retention of knowledge and enhanced clinical reasoning [8], [9], [10], [11], [12]. VPs widely defined as *“an interactive computer simulation of real-life clinical scenarios for the purpose of medical training, education, or assessment.”* [8] have been introduced in medical education more than 40 years ago [13]. The use and implementation of VPs in the fields of veterinary medicine has increased constantly, however the usage of VPs for senior students outweighs the usage in the preclinical years [14], [15], [16].

In 2012, teachers of biochemistry and physiology from veterinary faculties in Hannover, Budapest and Lublin, together with technical and didactical experts, started to establish an international and interdisciplinary consortium co-funded by the EU to launch the vetVIP (veterinary virtual patients)-project [17]. The members of the consortium were deliberately selected. These three participating universities differ in the level of experience and state of exposure to virtual patients. The goal was the joint development of a product that worked for all partners. The consortium was composed of scientists, teachers, educationalists and veterinarians with the common goal of improving teaching and learning of basic sciences in veterinary education. This working group decided to conduct a project similar to successful projects in medical education working with VPs [18], [19], [20], [21]. Furthermore, this project aimed to contribute to the recommendations of the European Association of Establishments for Veterinary Education (EAEVE) to exchange information and teaching staff as well [22]. In addition, this project was conducted to increase student’s interest in basic sciences using integrative and innovative learning tools to promote more meaningful and effective learning.

The aims of the vetVIP-project and research objectives of this study were:

1. To investigate whether it is possible to introduce VPs to veterinary basic sciences simultaneously at 3 different universities.
2. To evaluate students’ and authors’ perceptions towards utilization, acceptance and implementation of VPs alongside to traditional basic science education.

## 2. Methods

### 2.1. Setting

The vetVIP-consortium created computer-based learning material for teaching in basic sciences, using the case-based, multimedia learning and authoring system CASUS [23], [24], [25]. All members of the consortium were already experienced in e-learning approaches and reflected complementary scientific and educational competencies. In Hannover virtual patients in the CASUS-system had been used since 2005 and acceptance had already been evaluated [14]. In Lublin, the system CASUS was introduced in the subject chemistry in 2011 [26]. In Budapest, CASUS was newly introduced within the vetVIP-project. Each faculty generated 5 VPs in English and additionally translated the total number of 15 VPs to the respective national language. Content and learning objectives of all 15 VPs were discussed and chosen by mutual agreement. For each VP, an authoring team of scientists and corresponding clinicians were assigned. In order to guarantee a uniform design of all 15 VPs, guidelines for the authors were prepared and distributed. Prior to the distribution to students each VP was reviewed didactically and technically by collaborating experts from the E-Learning Department at the University of Veterinary Medicine in Hannover and from CASUS software developers, the Instruct AG in Munich. A quality of content review was performed mutually by all participating partners.

#### 2.2. Study design 

**Figure 1:**
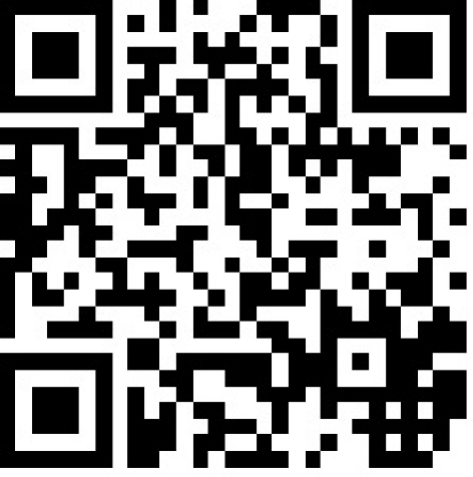
QR-Code YouTube Screencast

Students in their second year of veterinary study from all three universities were selected as participants for the first trial of VPs.The study population consisted of 795 veterinary undergraduate students in total (Budapest n=311; Hannover n=268; Lublin n=216). Students and their corresponding biochemistry teachers were informed and invited via e-mail and announcements, to use the VPs as an optional learning material alongside of regular courses. For the performance of the VPs no extra spare time was given. VPs were offered as independent, extracurricular study. In the winter semester 2013/2014 the 15 VPs were made available for the study cohort in an online CASUS-course. Students were invited to register for CASUS via e-mail. A YouTube-screencast was provided to illustrate the easy access and process of self-registration to CASUS, scan Figure 1 (Fig. 1) [27] for viewing.

The case usage was automatically registered in the CASUS system.

During the examination periods an anonymous online-survey was sent via e-mail to students from Hannover and Lublin. For answering the evaluation-survey, performance of cases was indispensable, so the survey was not sent to students from Budapest.

#### 2.3. Potential sources of methodological bias

As already described, the different level of implementation state of CASUS at all three locations must be considered. Furthermore, students were able to recognize the authors and location of origin within each VP. Due to delay in translations and the reviewing process of the VPs, Lublin decided not to make available all cases in different languages for their students. The beginning and end of the biochemistry courses and the corresponding final exams differed in time pattern. The evaluation-survey was only sent to students from Hannover and Lublin, as participation in Budapest did not require any evaluation.

#### 2.4. Data collection

Quantitative data, such as number of registered students, number of sessions, time spent per VPs, time spent per card of a VP, completeness and success rate of sessions were automatically registered and analysed by the integrated statistical software of CASUS and exported as user-log-files.

The online evaluation-survey was active for one month and the link was sent per e-mail to students from Hannover and Lublin several times as a reminder. The surveys obtaining student opinions about the quality of the virtual patients were sent during examination periods. In the anonymous online evaluation-survey, using the survey tool SurveyMonkey, students were asked to reflect on their experiences with VPs in veterinary basic sciences. The first part of the survey contained four questions regarding students’ profiles, such as university, semester of study, gender and age. The second part referred to the 15 VPs, asking which cases were processed and stating the favourites. Questions relating to the evaluation of the learning experience were displayed in the third part. 14 statements and one free text comment, extracted and modified from a validated evaluation tool kit [21], covered four categories: coordination, authenticity, learning effect and overall judgement. The questions were in multiple choice and scaled-response formats (6-point Likert scale: 1. strongly agree, 2. agree, 3. somewhat agree, 4. somewhat disagree, 5. disagree, 6. strongly disagree). A 6-point Likert scale was chosen to have an even number of ratings in the scale to have respondents commit to either the positive or negative end of the scale. 

In order to investigate the opinion of the authors, quantitative data was exported as user-log-files from CASUS. For qualitative evaluation an online focus group discussion on various aspects of creating and using VPs and CASUS was performed with randomly chosen and invited authors of each faculty. The online discussion was led along a prepared guideline [25]. The session was recorded, transcribed and clustered afterwards. Statements, which emerged during the focus group, were used for the preparation of a SurveyMonkey questionnaire for all involved vetVIP-project members. Responses to statements were also assessed using a 6-point Likert-scale. The survey was active for one month and the link was sent to the authors and reviewers several times per e-mail as a reminder.

#### 2.5. Data analysis

The web-based survey tool SurveyMonkey [https://de.surveymonkey.com/] with its integrated statistic software was used for the design, distribution, collection and analyses of the surveys.

Adobe® Acrobat® Connect™ Pro virtual classroom system of the company Adobe distributed via the German Scientific Network (Deutsches Forschungsnetz, DFN) was used for the conducted focus group. Included in this system is Voice-over-IP (VoIP), audio-communication and video-streaming and a browser- and flash-based dynamic work-surface with chat, whiteboard and other presentation possibilities.

User-log-files exported from the software CASUS, developed by the AG Medizinische Lernprogramme at the LMU were used for statistical analyses.

Further statistical analyses were carried out using SAS® software, version 9.3 (SAS Institute, Cary, North Carolina, USA). 

The observed distribution of the sums of performed case sessions within one group was compared with the expected distribution that students would perform cases originating from each university equally using the Chi-Square test for specified proportions.

These analyses aimed to interpret the student’s choices and reflect their preferences of case selection and usage.

Comparisons of statements ranked by students from the universities in Hannover and Lublin regarding the distributions of the Likert-scores were conducted by Fisher’s exact test.

In the same way, the three universities were compared regarding the answers of the authors.

## 3. Results

### 3.1. Virtual patient design

**Figure 2:**
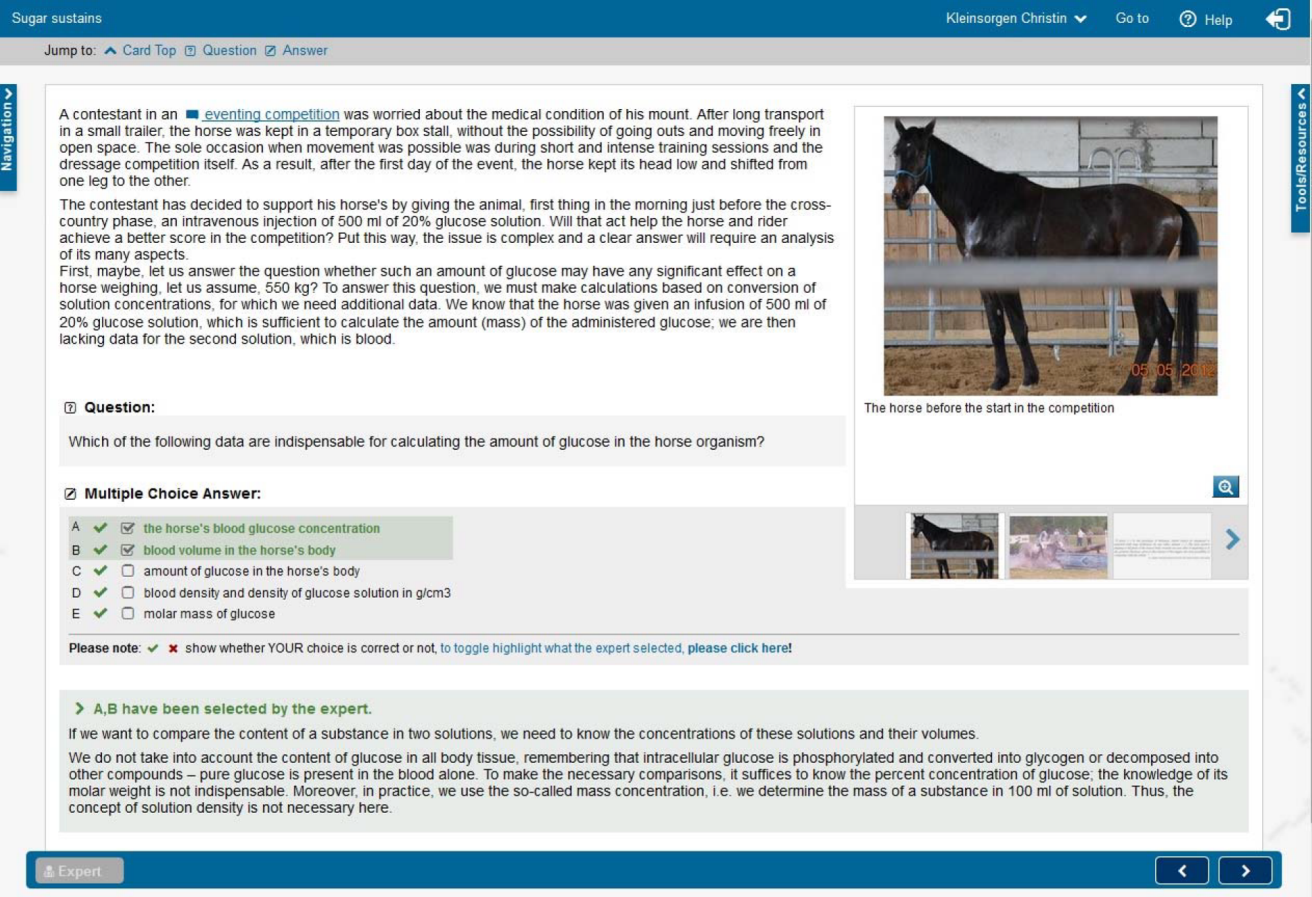
Screenshot of CASUS in player mode, card of case “Sugar sustains”

After 3 months of training 15 VPs were designed, reviewed and partly translated to English, German, Hungarian and Polish within 9 months. Each case consisted of 15-20 cards. Each card contained a text-field, media-files, one question, answer-choices and an answer-comment as immediate feedback (see Figure 2 (Fig. 2)).

On average, a virtual case contained 24 multimedia-files (images, videos, graphs, tables) and more than 13 questions of various answer-types (multiple choice, free text, underline, sorting, mapping etc.). Furthermore, expert comments with more detailed information, hyperlinks and PDF files referring to further literature were attached. A short introduction to all vetVIP-cases is available on the vetVIP-homepage [http://www.vetvip.eu/?q=de/home].

#### 3.2. Quantitative data obtained automatically in CASUS

##### 3.2.1. Authors

Throughout the editing and reviewing-process of the first 15 VPs, 15 experts had access to the installed vetVIP review course. The experts processed 90 sessions with an average of 34.5 minutes and reviewed the 15 cases technically, didactically and qualitatively using a prepared script for each review. In October 2013, 15 VPs were approved for publication for students in CASUS. However, not all translations have been completed and approved. Further minor revisions were made even after publication in the system. Nevertheless, Lublin decided not to make all 15 VPs available to their students in English and Polish.

##### 3.2.2. Students

In total, 391 (49.2%) of invited undergraduate students (N=795) of the universities in Budapest, Hannover and Lublin registered in the system CASUS. The registration rate was in Budapest 3.9% (12/311), in Hannover 75.4% (202/268) and in Lublin 81.9% (177/216). The percentage of students really performing in CASUS was in total 46.0% (366/795), in Budapest 1.0% (3/311), in Hannover 74.3% (199/268) and in Lublin 75.9% (164/216). In Hannover 173 (87%) female and 26 (13%) male students participated and in Lublin 129 (79%) female and 35 (21%) male students performed VPs.

In the following analysis, results from Budapest were only partially taken into account due to the very low participation and because no case was processed completely.

In total, 3455 started sessions by students were recorded. 164 students from Lublin started 1589 sessions and completed 1197 sessions successfully. On average, each student performed more than 9 sessions (mean average: 9.65), with an average time of 35.58 minutes (minimum: 1.07 minutes; maximum: 53.73 minutes) spent per session. In Hannover 199 users started 1869 sessions and completed 1517 sessions successfully, reporting an average number of 9.39 sessions per student. On average, students from Hannover spent 31.20 minutes (minimum: 11.83 minutes; maximum 49.49 minutes) per session.

**Figure 3:**
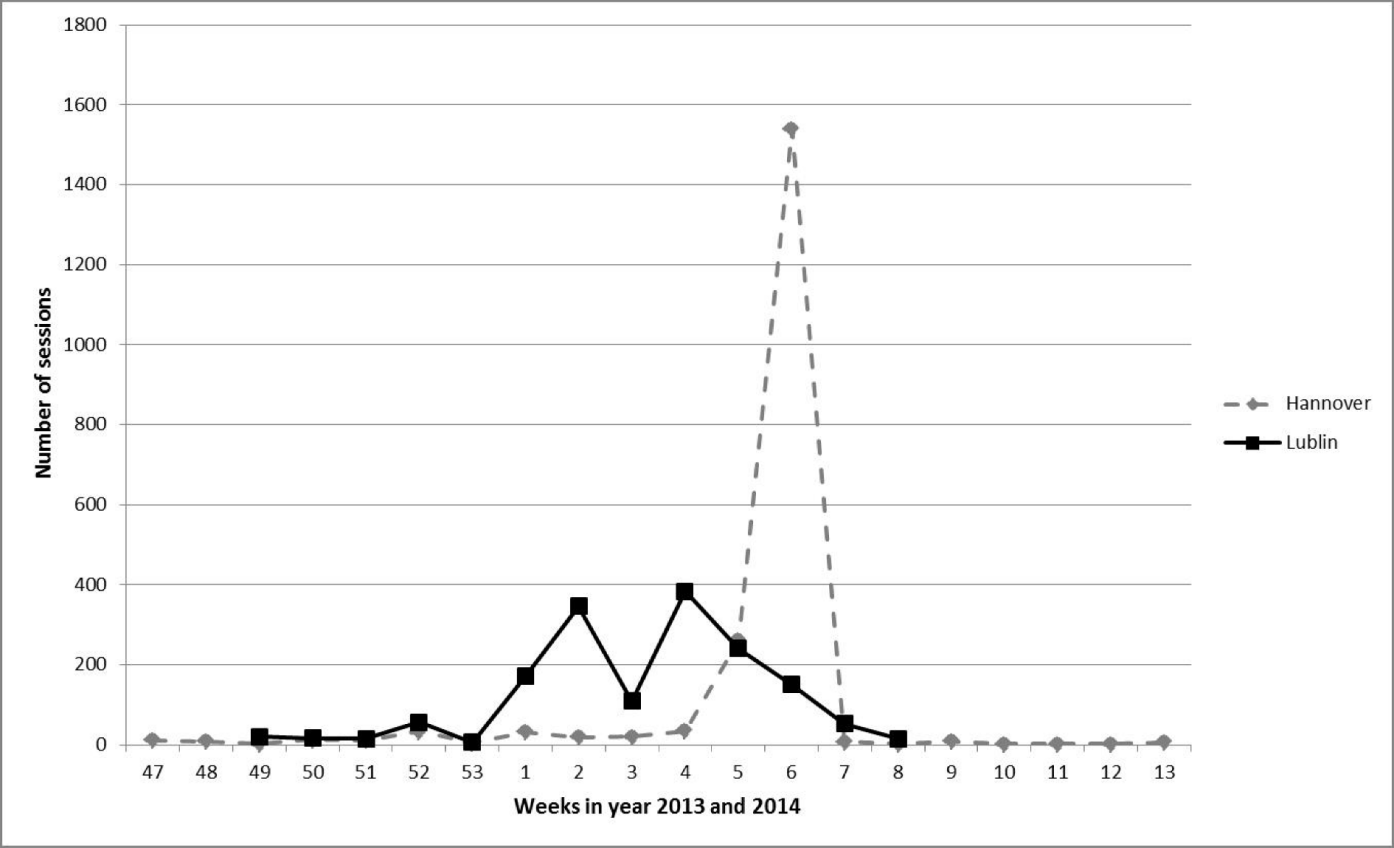
Numbers of started sessions per week

The utilization of VPs is illustrated in Figure 3 (Fig. 3) showing number of sessions per week in Hannover and Lublin. In Hannover the highest peak of utilization with 1540 sessions was in week 6 of year 2014. In Lublin the highest peak with 383 sessions was in week 4 of year 2014.

**Figure 4:**
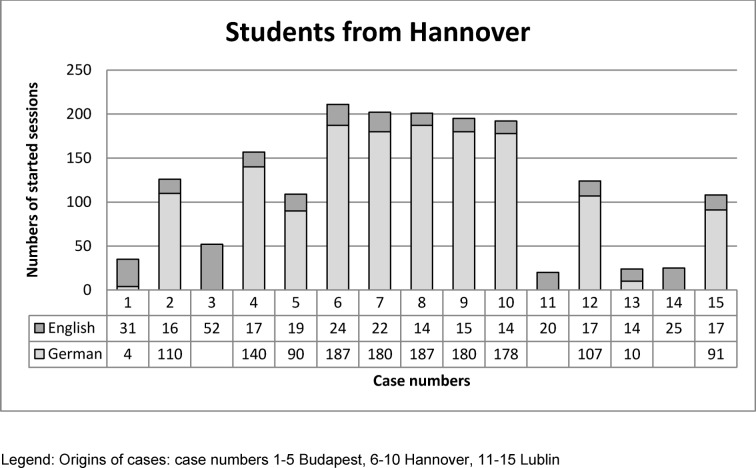
Numbers of started sessions per case of students from Hannover in native language or English.

**Figure 5:**
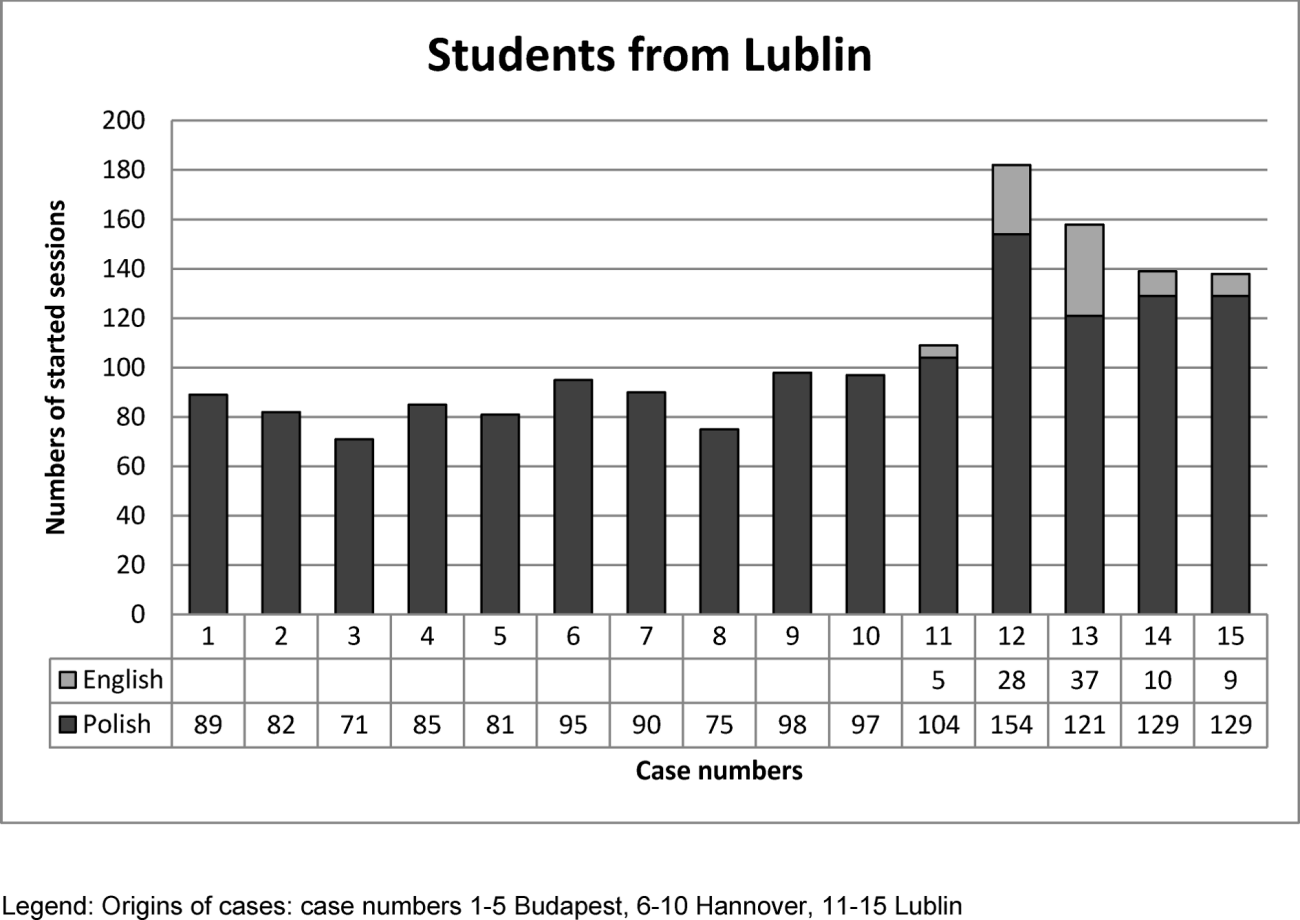
Numbers of started sessions per case of students from Lublin in native language or English.

The numbers of started sessions per case are illustrated in the following Figures 4 (Fig. 4) and 5 (Fig. 5).

Students from Hannover performed more cases originating from Hannover, than from Budapest or Lublin. The usage by students from Hannover of cases created in Budapest or Lublin was observed evenly distributed. Also, they performed more case sessions in their native language (German), when English version was offered, too. No differences in recorded case sessions in English by students from Hannover with regards to case origin were observed.

The case usage by students from Lublin show similar findings. In general, more cases originating from Lublin were performed. When English and Polish version of the case was offered, more sessions were performed in Polish.

The distributions of the sums of observed sessions are shown in Table 1 (Tab. 1).

According to case origins (Budapest, Hannover, Lublin,) an equally distributed case usage was expected (test percentage 33.33% each). In Hannover 12 VPs were offered in English and German language (see Figure 4 (Fig. 4) and Column 1 in Table 1 (Tab. 1)). So if equal distribution regarding to origins was estimated, expected test percentage with 4 cases from Budapest was 33.30%, 5 cases from Hannover 41.70% and 3 cases from Lublin 25%. The Chi-Square test for specified proportions revealed that the frequencies differ significantly (p=<.0001). Students from Hannover performed more cases created in Hannover. Furthermore, in Hannover only cases 2 and 5 from all 3 locations were offered in English and German (see Figure 4 (Fig. 4) and Column 2 in Table 1 (Tab. 1)). With expected test percent of 33.33 % per origin, students from Hannover performed more cases created in Hannover (p=<.0001).

In Lublin all 15 VPs were offered in Polish language (see Figure 5 (Fig. 5) and Column 3 in Table 1 (Tab. 1)). With expected distribution of equally performed case session regarding the origins (test percent=33.33% each), students from Lublin performed significantly more cases created in Lublin (p=<.0001).

#### 3.3. Evaluation surveys

##### 3.3.1. Students

In Hannover and Lublin altogether 176 from 484 invited students completed the online evaluation-survey (36.36%). Out of 118 started surveys from Hannover, a total number of 90 completed surveys could be rated (33.58% of the semester). The participants were with 82.5% female, 17.5% male, on average 21.87 years old. From the 116 started surveys in Lublin 86 were valid (39.81% of the semester). In this group were 73.3% female and 26.7% were male, averaging 20.9 years old.

**Figure 6:**
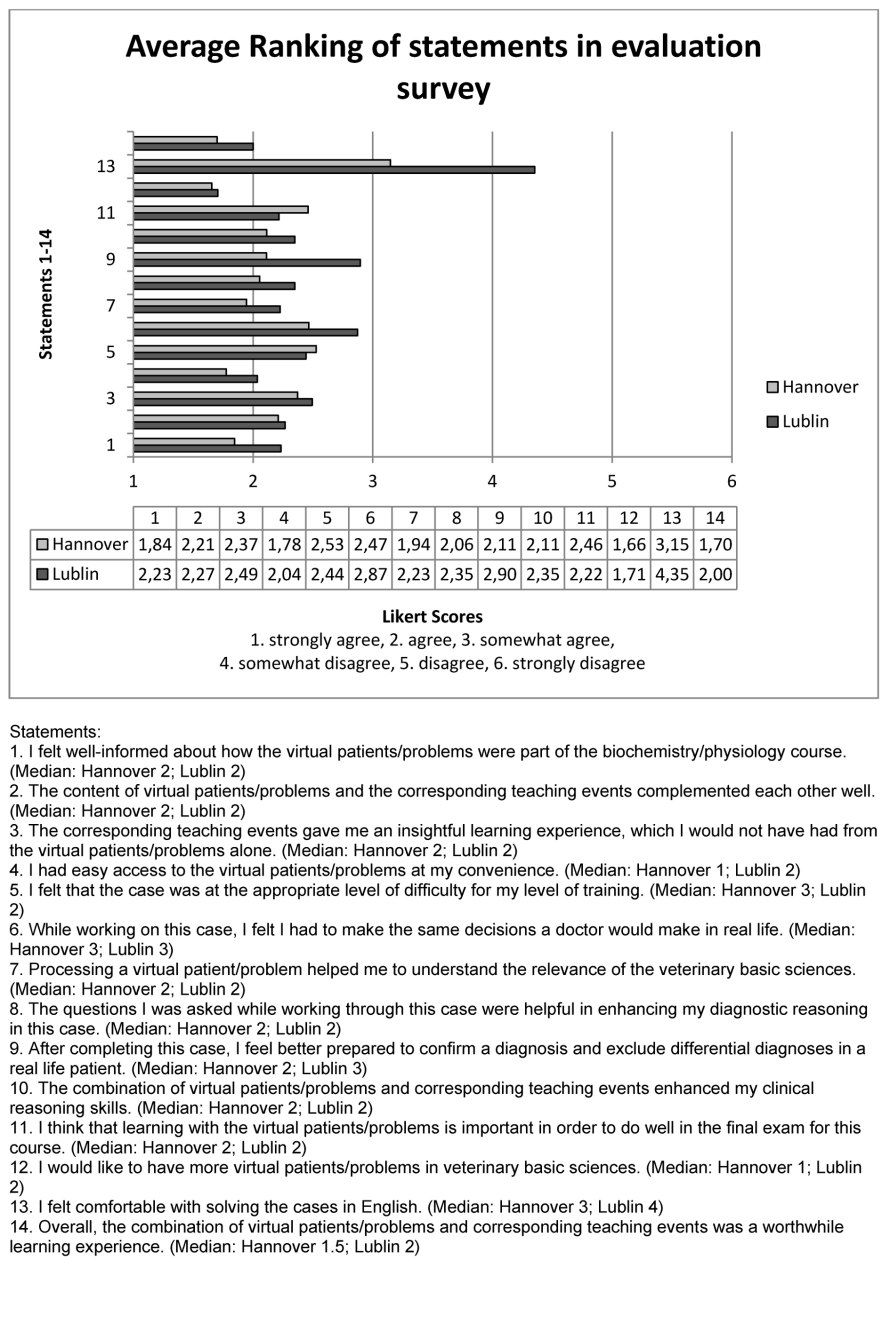
Evaluation survey: Distribution of student’s ratings of statements 1-14

Answers of students from Hannover and Lublin have been compared for each statement. Results show that Statements 1, 7-9, 11, 13-14 were ranked with statistically significant differences in distribution of the scores between Hannover and Lublin (see Figure 6 (Fig. 6)).

Out of 175 rated responses from Hannover and Lublin 170 students, (96.59%) agreed with the statement 14, that overall the combination of virtual patients/problems together with corresponding teaching events was a worthwhile learning experience.

The reaction of students mostly differed on the statement 13 “I feel comfortable to work through cases in English.” Students from Hannover agreed (mean average 3.15; median 3), whereas in Lublin students more likely disagreed (mean average 4.35; median 4).

In Hannover 15 extra free-text comments were given and in Lublin 10 comments. In total, 11 comments were positive statements. e.g.: “Thank you very much! An exciting, creative and very helpful way of teaching!”; “I am very grateful that the CASUS cases were created and would be glad if more would be done in future”; “I like to learn with tutorials. Also, I think it's good that some cases are written very funny. Learning makes fun like this :)”; “Very interesting way to transfer knowledge. With this form of learning, I noticed a sense of science in biochemistry and I realized that, however, it is needed in the study :)”.

4 comments addressed the level of case difficulty such as: “It seems to me that the course can be a little difficult for the students of the second year. But to me, as a student of the third year, the questions did not make much of a problem.”

10 comments addressed the demand for addition of more synonyms for free text answers or cloze tests, or the hint to include more special characters for the polish language.

##### 3.3.2. Authors

Altogether seven authors attended the two hours lasting online focus group (3 from Budapest. 2 from Hannover. 2 from Lublin). Main topics along the guideline for the discussion were the use of the CASUS-System, creation of cases, technical devices, communication and support, acceptance and the declaration of good cases. After clustering the transcript of this discussion, results were used to provide the evaluation-survey for the authors and reviewers.

The evaluation-survey was sent to all participating vetVIP-partners (i.e. first-authors and reviewers). 23 persons started the survey, of which 21 response counts were validated. Persons from the universities in Budapest, Hannover and Lublin as well as from the E-Learning Department in Hannover and the Instruct AG participated. 10 persons were disclosed as authors, 7 as first-authors and reviewers and 4 as reviewers only. On average each participant created more than 5 cases with the CASUS-System and reviewed more than 12 vetVIP-cases. An estimated time for the creation of a whole case was generally more than 30 hours (hours per week: minimum 5; maximum >50). Authors repeated having worked 4-5 weeks on average per case (weeks: minimum 1; maximum 10). The opinion about from which utilization rate among students the creation of cases is worth it differs from 20% to 80% (on average 48%).

In the second part of the survey, the authors and reviewers ranked statements using a 6-point Likert scale. According to exact Fisher-test in the response grid, 3 out of 47 statements were rated significantly different by authors grouped by the 3 locations Budapest, Hannover and Lublin. All participants agreed that cases should be used as supplementation to selected lectures or practical classes, but one specific case cannot replace one lecture about that topic. Further they agreed that cases should always be available for students and non-mandatory. Concerning the use of CASUS, they strongly agreed that cased based learning motivates the students and that they are more willing to learn using clinically relevant material. According to post-course comments of students and the evaluation, the authors believe that with CASUS students understand some mechanisms and topics better. Case-based learning can show students the importance of basic sciences for solving clinical problems. Authors repeat that learning with CASUS can improve the way of diagnostic thinking. Advantages of CASUS are the time and place flexibility, the opportunity of training logical and diagnostic thinking, the motivation for learning and the chance to deepen student’s knowledge. The authors and reviewers agreed that for the creation of cases with clinically relevant topics, the interaction between basic scientists and veterinarians is crucial. They state that a good case means a close correlation of biochemical and physiological theory with a realistic patient. All authors mentioned that feedback from students during the creational process helped to create good cases. All participants agreed that a content-related, technical and didactical review of each case is necessary. They all positively ranked the statements “I like creating cases”, “Use of e-learning in veterinary basic sciences is beneficial”. Overall, they strongly agreed that the combination of basic theory with clinical cases is useful (mean average=1.38), that the use of case-based learning material for undergraduate students is useful (mean average=1.50), more colleagues should use CASUS (mean average=1.60), and more cases should be created and used (mean average=1.40).

## 4. Discussion

As Poulton and Balasubramaniam reviewed in 2011: *“VPs have become more attractive, more available and easier to create… They have begun to penetrate distinct areas of the core of the undergraduate medical curriculum, driven both by students-teacher interest and by recognition of their pedagogic value.” *[28].

The teaching of basic sciences, in particularly the subject biochemistry, has been critically reviewed long ago, but whether learning objectives were achieved is still under debate [2], [3], [4], [5], [6], [7]. Also, the relevance of the basic science content in undergraduate education has already been argued [29], [30]. Jason highlighted already in 1974 *“that content is best learned and evaluated in the same context in which it will later be put to use, and problem-solving skills are best learned and evaluated in the specific situation in which they are to apply”* [29].

The goal to create VPs with biochemical and physiological background and combining theoretical with clinical knowledge was achieved. Furthermore, students get trained to solve problems similar to their future professional activity. Integrative clinical case scenarios encourage active learning and the development of higher order thinking skills.

At all 3 participating universities, the education in basic sciences is mainly teacher-centred, i.e. lecture-based with few practical courses. In Hannover and Lublin, the education in the subject biochemistry lasts for 1 year (2 semesters). In total there are 60 hours of lectures in Lublin, 84 in Hannover; and 90 hours of practical classes in Lublin and 28 hours in Hannover. As most of the basic science teachers engaged in this study are not veterinarians, the learning objectives and content taught often does not seem clinically relevant to students. In general, students consider biochemistry mainly as a subject with a myriad of chemical structures and complex pathways unrelated to their future success as a veterinarian.

The format of examination in the subject biochemistry differed at all 3 participating universities. In Hannover there are 2 written exams (Multiple-Choice format). In Budapest and Lublin, students have to pass an oral examination. All 3 locations assess in the appropriate native language (i.e. Hungarian, German and Polish). 

None of the participating biochemistry or physiology departments have an official national catalogue or an agreement for a checklist of learning objectives. Hence, the content of the lectures or practical courses differed at all 3 universities.

Due to use of mainly lecture-based basic science education at the participating universities, the positive evaluations must be scrutinized more closely. Based on the data collected, it is not possible to relate their positivity explicitly to the specific type of learning intervention. Students might respond positively to any increase in innovative teaching methods. Here further investigations for clarification are needed.

Within the vetVIP-project the introduction of VPs into traditional curriculum at the universities in Budapest, Hannover and Lublin was meant to be an educational vehicle to link basic science with veterinary clinical medicine. The replacement of real patients was never intended during the process of creation of the vetVIP-cases. Their usage was considered as an optional opportunity to practice clinical problem-solving and as an additional tool to learn and understand [31]. The VPs were offered as supplementary teaching units and were only used and advertised by some teachers as blended learning scenarios, but not mandatory. So an actual blended use was not conducted.

In order to provide VPs with an appropriate level of difficulty both the content, adjusted to the level of knowledge as well as the linear predetermined navigation, were deliberately chosen. According to the principles of virtual patient designs [32], a linear navigation and scaffolding are preferred from students [33], but are not always realistic. Furthermore, the preferred immediate specific feedback on each decision students made was delivered in answer- or additionally in expert-comments.

While usage and acceptance in Lublin and Hannover was recognizably high (>70%), the participation rate in Budapest was below 1%. Possible explanations include different types of advertising for the optional courses and the level of implementation and awareness of the system CASUS itself [14], [26].

In addition, an increase in motivation to learn basic sciences can be considered from surveyed students and authors. Roger Heutschi (2003) stated in his review of the system CASUS the criteria ‘motivation’ with an outstanding rating, emphasizing the intuitive user- and author-interface, the flexibility in content, time, place and pace of usage. In our study all of those criteria have been positively rated in the focus group and evaluation-surveys of both authors and students as well.

Highest peaks of usage in Lublin and Hannover were observed during pre-examination periods. In Hannover, the peak, with more than 1500 registered case sessions per week was one week prior to the final biochemistry exam. In Lublin, two peaks were seen correlating with the staggered final exam period in biochemistry. These observations support the well-known statement that “assessment drives learning” and serves as an important motivation-factor for students [34]. In our study, the questions used in the VPs covered educational objectives tested in examinations at all three locations. However, format and phrasing of questions differed and only some similarities were recognizable. One comment given by a student from Hannover in the evaluation-survey emphasizes this as well; it was stated that the aforementioned relevance of questions used in the cases for the examination has lured him to use the VPs as exercise before the exam. Similar findings have been published by Hege et al. defining this phenomenon as “exam strategy” for higher percentages in case usage [24].

Under the aspect of "assessment drives learning", it is not striking that students from Hannover more frequently performed cases in German designed by the biochemistry-department of Hannover (p=<.0001), as it seems more likely to be relevant for the exams in Hannover. As authoring teams mutually agreed on content, this assumption of students deviated from actual situation. Students were able to distinguish the origin of the cases within the name of the case. For a reliable result of student’s preferences in case selection, it should have been a prerequisite not to show the origin of each case within the name. Also noticeable in Hannover and Lublin, is the higher usage of cases in the native language, when English version was additionally offered next to the Polish or German version. The limitation in availability of the cases occurred due to delay in the translation- and the reviewing-process. Although there was mutual agreement on the strategy of implementation of the VPs, the realization differed at all three locations.

According to our experiences, the participation-rate in the evaluation-survey with more than 36% valid responses (Hannover=33.58%; Lublin=39.81%) is a fairly good response count of a voluntary online-survey. It should be noted that there is a possibility of an indirect positive selection; it may be that only the most interested students participated in this voluntary survey.

Throughout the whole evaluation-survey assessed by students from Hannover and Lublin positive statements were rated. Except for one disagreement about working through cases in English all categories (coordination, authenticity, learning effect, overall judgement) were ranked positively. In general students from Hannover reviewed each statement with slightly higher agreement.

In the evaluation-survey students from Hannover mostly agreed on the question “I feel comfortable to work through cases in English.” (mean average 3.15), whereas in Lublin students on average somewhat disagreed (mean average 4.35). It should be noted that only 7 successfully completed cases from Polish students in English were registered, so here only estimated opinions were expressed, not opinions based on experiences. In conclusion it still can be stated that students seem to prefer optional learning-material in their native language, respectively in the language of examination.

On average the statements given by the authors during the focus group and the evaluation-survey emphasized and even boosted this positive feedback by students. The increase in motivation to produce and to use more case-based teaching and learning in biochemistry and physiology were mentioned. The 3 out of 47 statements ranked differently by the authors and reviewers could also be explained by the statistical error of the first kind. As differences in the results of these specific 3 statements are not noteworthy, these are neither illustrated in this manuscript nor further investigated. 

During the focus group interviews, various aspects of case creation and technical problems were critically discussed. Criteria such as the time and financial frame for creation of cases were discussed, like already published by Ehlers et al. before [35]. Another focus was on the usage of appropriate media. Some authors stated difficulties in taking or finding good pictures, always considering the data-protection law. In other studies the mean time spent for the creation of VP’s has been described with 20-80h [21], which corresponds with the statements of our authors.

The high utilization rates in combination with the positive feedback from students and authors underline the attractiveness and practicability of VPs in veterinary basic sciences. Both authors and students agreed, that overall the combination of virtual patients/problems and corresponding teaching events was a worthwhile learning experience. A great acceptance was shown by high utilization rates and an increase in motivation was stated from authors and students. The results of this study are limited by the small size of the case sample (N=15), and thus should be viewed as preliminary observations. A larger sample size of cases, preferably obtained from multiple student cohorts at the 3 participating or even more universities, would be needed to confirm and generalize our findings.

## 5. Conclusions

In conclusion, veterinary virtual patients in basic sciences can be used for the presentation of integrative clinical case scenarios, which encourage active learning and the development of higher order thinking skills.

Throughout the vetVIP project, 15 VPs have been successfully created, whereas the success of the implementation-process differs at all 3 participating universities. The positive feedback by students and authors lead to the development of further 15 VPs, so by now 30 vetVIP-cases in veterinary basic sciences were created and reviewed.

Above all, this project has provided an extraordinary experience for the participating students and teachers in veterinary basic sciences as they reflected on their own learning and teaching. Teachers were engaged to increase the student’s motivation for learning. Furthermore, the project provided the opportunity for integrative and innovative learning material that can be used alongside to current teaching and learning. By now, several European veterinary universities expressed their interest in using and sharing the VPs created within the vetVIP-project. As a result, we believe that the outcomes of the project can be sustained and expanded.

Plans for the future include further studies on the learning effects and the ability of knowledge transfer. Additionally the range of VPs and expansion of the offer to other subjects shall be considered. To take account of the sustainability of this work with VPs in veterinary basic sciences one possibility could be to provide open access to the vetVIP-course, including creative commons licensed cases.

## Acknowledgements

Scientific work of Polish partners was co-financed by funds from Polish Ministry of Science and Higher Education (years 2012-2014) assigned for the realisation of international project vetVIP.

Special thanks to the students, veterinarians and educationalists, who volunteered to participate in and helped with project activities.

The authors thank Prof. Duncan Ferguson for proof reading of the English version.

Members of the vetVIP-Consortium:

University of Life Sciences in Lublin: Marta Kankofer, Zbigniew Gradzki, Witold Kedzierski, Jacek Wawrzykowski, Marta Wojcik, Marta Giergiel, Michal Danielak, Marek Szczubial, Wojciech Lopuszynski, Ewa Sobieraj

Szent Istvan University in Budapest: Bartha Tibor, Mandoki Mira, Tóth István, Somogyi Virág, Jócsák Gergely, Kiss Dávid Sándor, 

University of Veterinary Medicine in Hannover: Hassan Y. Naim, Maren von Köckritz-Blickwede; Graham Brogden, Katja Branitzki-Heinemann, Sucheera Chotikatum, Lena Diekmann, Eva-Maria Küch, Helene Möllerherm, Christin Kleinsorgen, Jan P. Ehlers

Instruct AG Munich: Martin Adler

## Funding

The vetVIP project (Use of virtual problems/virtual patients in veterinary basic sciences) was supported by an EU grant (526137-LLP-1–2012-1-PL-ERASMUS-FEXI, EU Lifelong Learning Programme).

## Competing interests

The authors declare that they have no competing interests.

## Figures and Tables

**Table 1:**
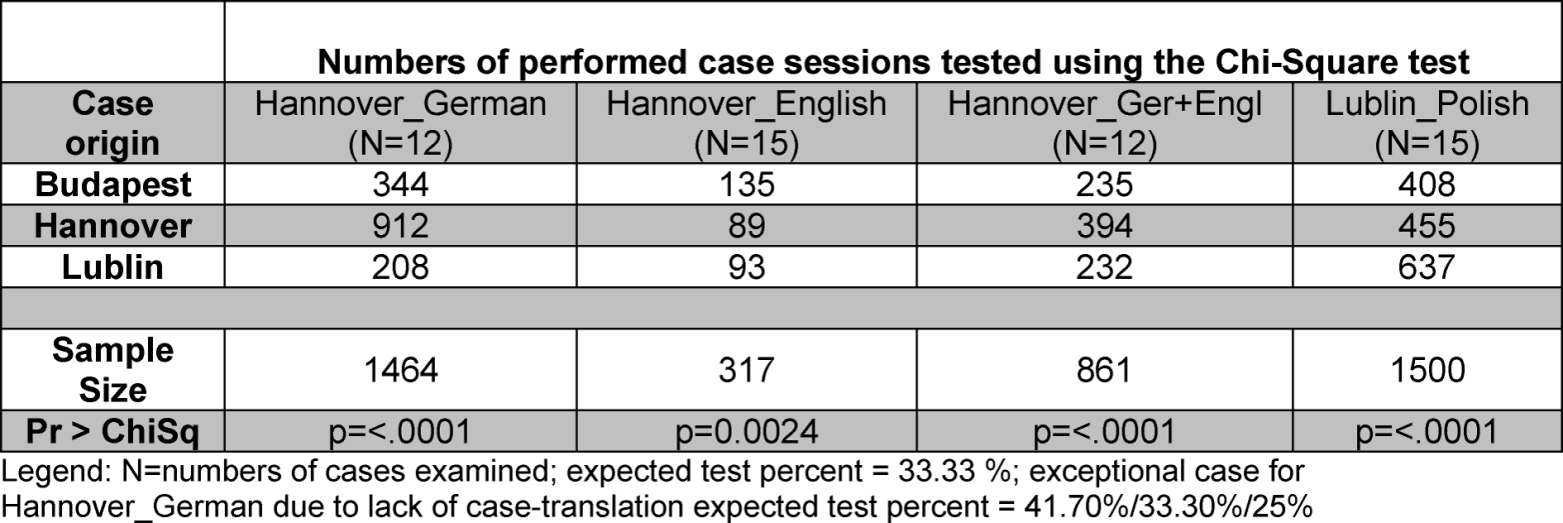
Comparison of frequencies of performed case sessions
